# Induction of specific T helper-9 cells to inhibit glioma cell growth

**DOI:** 10.18632/oncotarget.13981

**Published:** 2016-12-16

**Authors:** Haiyan Zheng, Baohua Yang, Dedong Xu, Wenbo Wang, Jie Tan, Liyuan Sun, Qinghua Li, Li Sun, Xuewei Xia

**Affiliations:** ^1^ Department of Neurosurgery, The Fourth Affiliated Hospital, Zhejiang University School of Medicine, Yiwu, 322000, Zhejiang, China; ^2^ Department of Neurosurgery, Guilin Medical University, Affiliated Hospital, Guilin, 541001, China; ^3^ Guangxi Key Laboratory of Brain and Cognitive Neuroscience, Guilin Medical University, Guilin, 541001, China; ^4^ Department of Neurosurgery, Hainan General Hospital, Haikou, 570311, China

**Keywords:** glioma, immunotherapy, staphylococcal enterotoxin B, interleukin-9, T helper-9 cell

## Abstract

The effects of Staphylococcal enterotoxin B (SEB) on regulation of immune response have been recognized; whether SEB can enhance the effects of immunotherapy on glioma remains to be investigated. This study tests a hypothesis that administration with SEB enhances the effects of specific immunotherapy on glioma growth in mice. In this study, a glioma-bearing mouse model was developed by adoptive transfer with GL261 cells (a mouse glioma cell line). The mice were treated with the GL261 cell extracts (used as an Ag) with or without administration of SEB. We observed that treating glioma-bearing mice with the glioma Ag and SEB induced glioma-specific Th9 cells in both glioma tissue and the spleen. Treating CD4^+^ CD25^−^ T cells with SEB increased p300 phosphorylation, histone H3K4 acetylation at the interleukin (IL)-9 promoter locus, and increased the IL-9 transcriptional factor binding to the IL-9 promoter. Treating CD4^+^ CD25^−^ T cells with both SEB and glioma Ag induced glioma-specific Th9 cells. The glioma-specific Th9 cells induced glioma cell apoptosis in the culture. Treating the glioma-bearing mice with SEB and glioma Ag significantly inhibited the glioma growth. In conclusion, SEB plus glioma Ag immunotherapy inhibits the experimental glioma growth, which may be a novel therapeutic remedy for the treatment of glioma.

## INTRODUCTION

Glioma is a common malignant tumor in the brain, accounting for approximately 50% of primary brain tumors. Despite rapid developments of surgical therapy, radiation therapy and chemotherapy in recent decades, the therapeutic effect on glioma is still poor [1]. One of the pathologic features of glioma is that forms microsatellites extensively in the normal brain tissue [2], which makes a great difficult to radically remove glioma surgically. Thus, other additional anti-tumor strategies may be carried out to enhance the effects of surgical or/and radiological therapies for glioma.

The immunotherapy has been employed in the treatment of cancer for years [3]. The discovery of cancer specific antigens (Ag), including exclusively or preferentially expressing in tumors, greatly potentiate the immunotherapy in cancer [3]. The major antitumor immune cells include T helper (Th)1 cells, Th2 cells, CD8^+^ cytotoxic T cells, natural killer (NK) cells and NKT cells, etc. These antitumor cells release cytokines to induce cancer cell death and thus inhibit cancer growth [4, 5]. Recent studies suggest that Th9 cells have high efficiency to induce cancer cell death. The major cytokine of Th9 cells is interleukin (IL)-9 [5, 6]. Given the multiple function of IL-9, Th9 cells participate in the pathogenesis of a number of diseases, such as cancer, allergic inflammation, and parasitosis [7]. The underlying mechanism by which IL-9 inhibits cancer growth is to be further investigated. The generation of Th9 cells in tumor-bearing subjects is unclear.

Staphylococcal enterotoxin B (SEB) is a superantigen; it is produced by Staphylococcal aureus. SEB has a unique capability in the activation of T cells via creating a link between T cell receptor and the MHC II molecule in dendritic cells (DC); it has been regarded as an immune regulatory agent [8]. Cumulative evidence indicate that SEB can induce IL-4 over production such as in skewed Th2 polarization [9]. Concurrent exposure to IL-4 and transforming growth factor (TGF)-β can generate Th9 cells [10]. Yet, whether SEB is associated with the Th9 cell development has not been investigated.

Based on the above information, we hypothesize that the co-presence of SEB and glioma specific antigens induces glioma specific Th9 cell development; the latter is capable of inhibiting glioma growth. In this study, we observed that SEB can induce the development of Th9 cells, which were capable of suppressing glioma growth in mice.

## RESULTS

### Immunotherapy of Ag/SEB induces glioma-specific Th9 response in the mice

**Figure 1:**
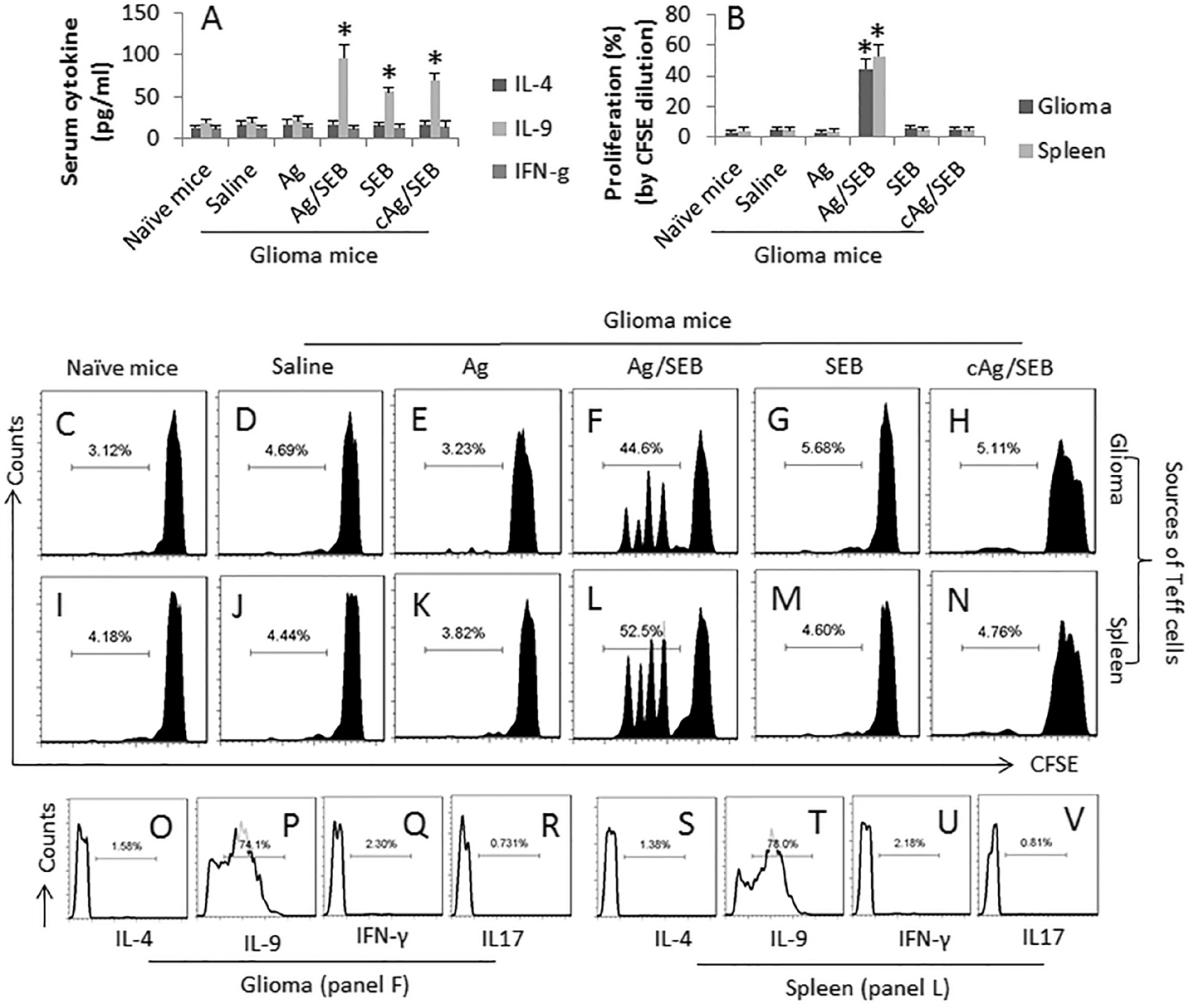
SEB facilitates glioma-specific Th9 response in glioma-bearing mice Glioma-bearing mice were treated with Ag (the GL261 cell extracts; 100 μg/mouse; cAg, the NG108-15 cell extracts, 100 μg/mouse; used as a control Ag) or/and SEB (2 μg/mouse; ip). Samples of serum, glioma tissue and the spleens were collected at sacrifice. (**A**) the bars indicate the serum levels of IL-4, IL-9 and IFN-γ (by ELISA). (**B**) the bars indicate the proliferation rate of Teff cells in the glioma tissue and the spleen (by flow cytometry). (**C**–**N**), CD4^+^ CD25^−^ T cells were isolated from the spleen and glioma tissue by MACS, stained with CFSE, and cultured with DCs in the presence of glioma cell extracts (5 μg/ml) for 3 days. The histograms show the representative results of Teff cell proliferation (the summarized data are presented in B). (**O**–**U**), the histograms show the frequency of IL-4^+^ cells, IL-9^+^ cells, IFN-γ^+^ cells and IL-17^+^ cells in panel F (O-R) and panel K (**S**–**V**) respectively. Each group consists of 9 mice. The sera from individual mice were analyzed separately; the data represent 3 independent experiments. Teff cells isolated from 3 glioma tissue were pooled to one sample (due to the small amount of cell number). The data of C-G represent 3 samples per group performed in one experiment. Teff cells isolated from individual mice were processed separately. The cell ratio in the proliferation assay is 5 × 10^4^ Teff cells: 1 × 10^4^ DCs. Phorbol-12-myristate-13-acetate (PMA; 20 ng/ml) was added to the culture to activate the Teff cells. Each group consists of 9 mice.

It is reported that IL-9 can inhibit cancer growth [11]. We wondered if the IL-9-producing cells could be induced in tumor-bearing mice by immunotherapy. To this end, we developed a mouse model with glioma cell subcutaneous transplantation. The mice were treated with the Ag/SEB immunotherapy. After sacrifice, the sera were analyzed by ELISA. The results showed that, comparing to control group, the serum levels of IL-9 were increased markedly in the Ag/SEB group; mice treated with either Ag alone did not show an apparent increase in the serum levels of IL-9. The serum levels of IL-4 and IFN-g were not changed significantly in all groups (Figure 1A). The results suggest that the Ag/SEB immunotherapy induces IL-9 production in the glioma-bearing mice.

**Figure 2:**
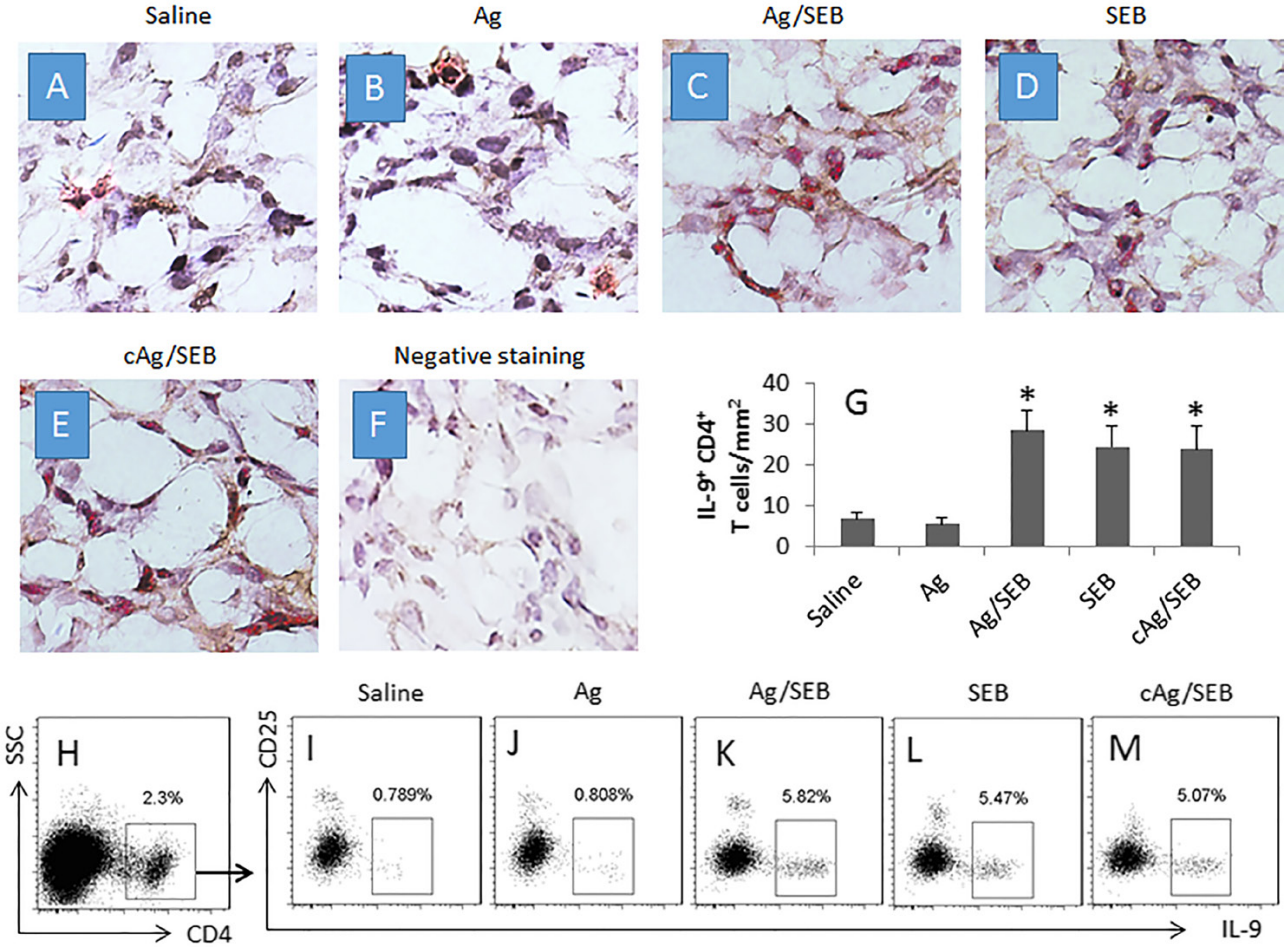
Counts of IL-9+ CD4+ T cells in glioma tissue (**A**–**F**) the photomicrographs show the IL-9^+^ (in red) CD4^+^ (in brown) T cells in glioma tissue from glioma-bearing mice. Panel F is negative staining controls (stained with isotype IgG). Image magnification: ×400. (**G**) the bars (mean ± SD; *p < 0.01, compared with the saline group) indicate the summarized data of panels A-F (averaged from 20 fields of each mouse). (**H**) CD4^+^ T cells were gated from the single cells of glioma tissue. (**I**–**M**) the gated plots indicate IL-9^+^ CD25^−^ T cells in the CD4^+^ T cells as shown in panel H. The data of bars are presented as mean ± SD. *p < 0.01, compared with the saline group. Each group consists of 9 mice. Samples from individual mice were processed separately. cAg is NG108-15 cell extracts, 100 μg/mouse; used as a control Ag.

We next took a further insight into the Th9 response in the mice. We isolated the effector T cells (Teff cells; CD4^+^ CD25^−^ T cells) from the glioma tissue and the spleen of mice. The Teff cells were cultured in the presence of the glioma cell extracts and DCs. As analyzed by flow cytometry, an Ag-specific Teff cell response was detected in mice treated with Ag/SEB. The rate of proliferating cells was significantly higher in the Ag/SEB group than the Ag group or the SEB group (Figure 1C–1N). Further analysis showed that the proliferated cells were mainly IL-9^+^, not IL-4^+^, or IL-17^+^ or IFN-γ^+^, in Ag/SEB group (Figure 1O–1U). The results indicate that the immunotherapy of Ag/SEB induces glioma-specific Th9 Teff cells in the mice. To corroborate the results, we treated the glioma-bearing mice with SEB plus an irrelevant Ag, the extracts from NG108-15 cells (another mouse glioma cell line). The results showed that no specific immune response was induced (Figure 1). In addition, IL-9^+^ CD4^+^ T cells were observed in the glioma tissue, which were more in mice treated with Ag/SEB, or SEB, or cAg/SEB than those treated with saline or Ag alone (Figure 2).

### SEB induces IL-9 expression in CD4^+^ T cells

**Figure 3:**
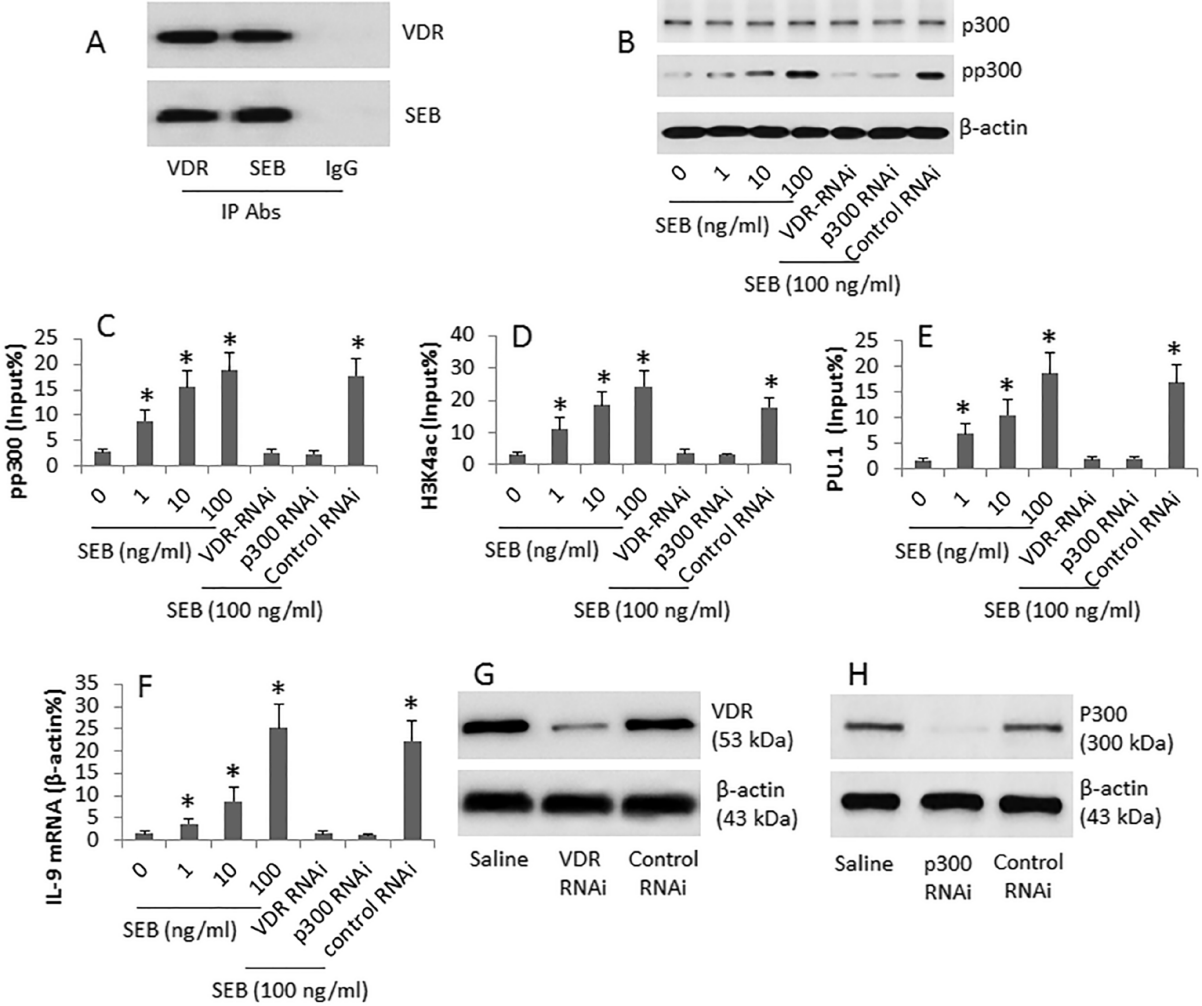
SEB regulates IL-9 gene expression in CD4+ T cells CD4^+^ CD25^−^ T cells were cultured SEB (at gradient doses as denoted) for 6 days. Cytosolic and nuclear extracts were prepared from the cells. (**A**) the immune blots indicate a complex of SEB and VDR (vitamin D receptor). (**B**) the immune blots indicate the levels of phosphorylated p300. (**C**–**E**) ChIP assay data; the bars indicate the levels of p300 (C), acetylated H3K4 (D) and PU.1 (E) at the IL-9 promoter locus. (**F**) the bars indicate the mRNA levels of IL-9 in the cytosolic extracts (by RT-qPCR). (**G**–**H**) the immune blots indicate the RNAi results of VDR (G) and p300 (H). The data of bars are presented as mean ± SD. *p < 0.01, compared with the dose “0” group. The data are representatives of 3 independent experiments.

VDR (vitamin D receptor) is involved in promoting p300 activities [12]; we wondered if SEB interacted with VDR to regulate p300 phosphorylation. To test this, we performed immunoprecipitation with antibodies of VDR and SEB. The results showed a complex of SEB and VDR was detected in GL261 cells extracts (Figure 3A). It is reported that p300 is involved in the IL-9 expression [13]. We then assessed the p300 phosphorylation in the CD4^+^ T cells after exposing to SEB in the culture. The exposure to SEB increased the p300 phosphorylation in the cells (Figure 3B). The activation of p300 in the target cells implies certain modulation may be induced in the chromatin to modulate target gene transcription [14]. Thus, we performed a ChIP assay with the cell extracts from the SEB-treated cells. The results showed that the pp300 levels (Figure 3C) and acetylated H3K4 (Figure 3D) were increased at the IL-9 promoter locus in a SEB dose-dependent manner. Since the histone acetylation provides an opportunity for transcriptional factor to access promoter [14], we then assessed the levels of the IL-9 gene transcriptional factor, PU.1, at the IL-9 promoter locus. The results showed that the significantly increase in the PU.1 levels was detected (Figure 3E), which was followed by the increases in the IL-9 mRNA (Figure 3F). To further corroborate the results, CD4^+^ T cells were treated with RNAi of VDR or p300, and then treated with SEB. Indeed, the expression of IL-9 was abolished by either VDR RNAi (Figure 3G) or p300 RNAi (Figure 3H). In addition, we also assessed the binding rate of pp300 and H3K4 at the promoter loci of IL-4, IFN-g and IL-17 in CD4^+^ T cells after exposure to SEB in the culture. The results showed no detectable effects of SEB on elevating the binding rate (Supplementary Figure S1 in Supplementary Materials). The exposure to SEB also did not alter the mRNA levels of IL-4, IFN-g and IL-17 in CD4^+^ T cells (Supplementary Figure S2).

### SEB generates Th9 cells *in vitro*

**Figure 4:**
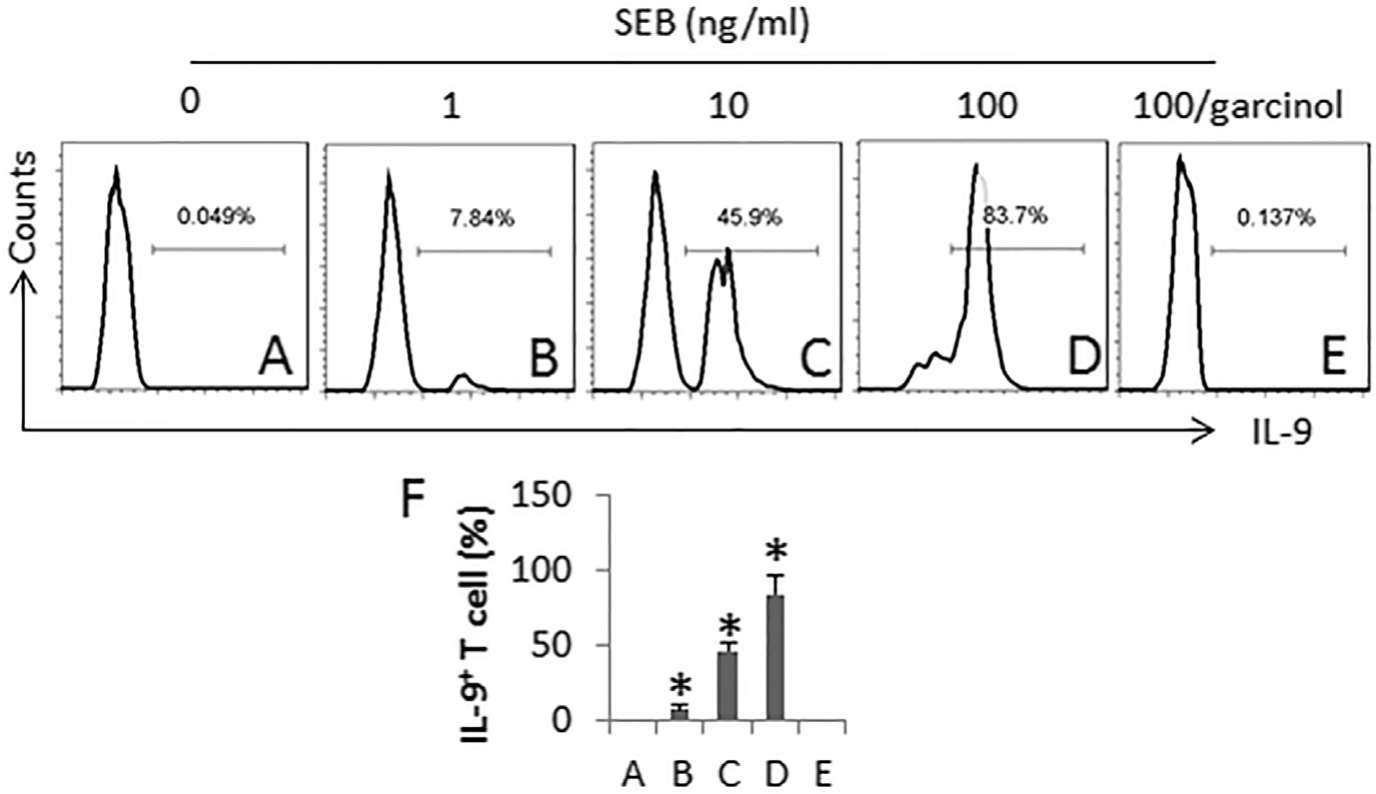
SEB induces Th9 cells CD4^+^ CD25^−^ T cells and DCs (T cell:DC = 5:1) were cultured in the presence of SEB (100 ng/ml) and IL-2 (10 ng/ml; used as a T cell activator) for 6 days. The medium and related agents were changed in every 3-day. The cells were collected at the end of the culture and analyzed by flow cytometry. (**A**–**E**), the histograms indicate the frequency of IL-9^+^ T cells. Garcinol (an inhibitor of p300; 1 μg/ml). (**F**) the bars indicate the summarized data of A-E (mean ± SD. *p < 0.01, compared with group A). The data are representatives of 3 independent experiments.

To further investigate the role of SEB in the induction of Th9 cells, we performed an *in vitro* study. CD4^+^ CD25^−^ T cells were cultured in the presence of SEB for 6 days. As assessed by flow cytometry, SEB markedly induced IL-9 expression in the T cells in a SEB dose-dependent manner, which was abolished by the presence of garcinol, a p300 inhibitor (Figure 4). The results demonstrate that SEB is capable of inducing Th9 cell differentiation.

### Exposure to SEB together with glioma cell extracts generates glioma-specific Th9 cells

**Figure 5:**
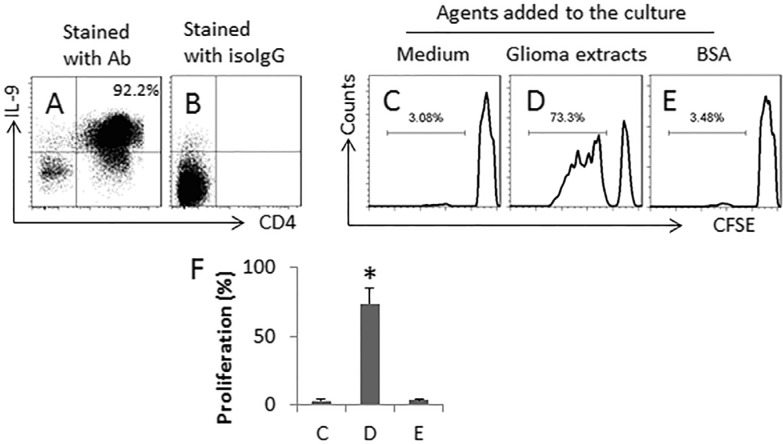
SEB and glioma cell extracts induce glioma-specific Th9 cells Th9 cells were generated from naïve CD4^+^ CD25^−^ T cells with the presence of glioma extracts (5 μg/ml) and DCs in the presence of PMA (20 ng/ml). (**A**–**B**) the dot plots show the frequency of Th9 cells (A); panel B is an isotype control. The T cells were collected and negatively selected by MACS, labeled with CFSE and exposed to medium, or glioma cell extracts, or BSA (a non-specific antigen, using as a control) in the presence of freshly isolated DCs (DC: T cells = 10^4^ cells: 10^5^ cells) for 3 days. The cells were analyzed by flow cytometry. (**C**–**E)**, the gated histograms indicate the proliferated T cells. (**F**) the bars indicate the summarized data of C-E. The data are presented as mean ± SD. *p < 0.01, compared with group C. The data are representatives of 3 independent experiments.

We next exposed CD4^+^ CD25^−^ T cells to SEB and glioma cell extracts in the culture in the presence of DCs for 6 days. More than 90% of the cell population was differentiated to Th9 cells (Figure 5A–5B). After exposing to glioma cell extracts (using as a specific Ag), more than 70% Th9 cells proliferated (Figure 5C–5F). The results indicate that exposure to SEB and glioma Ag can induce glioma specific Th9 cells.

### Specific Th9 cells induce glioma cell apoptosis

**Figure 6:**
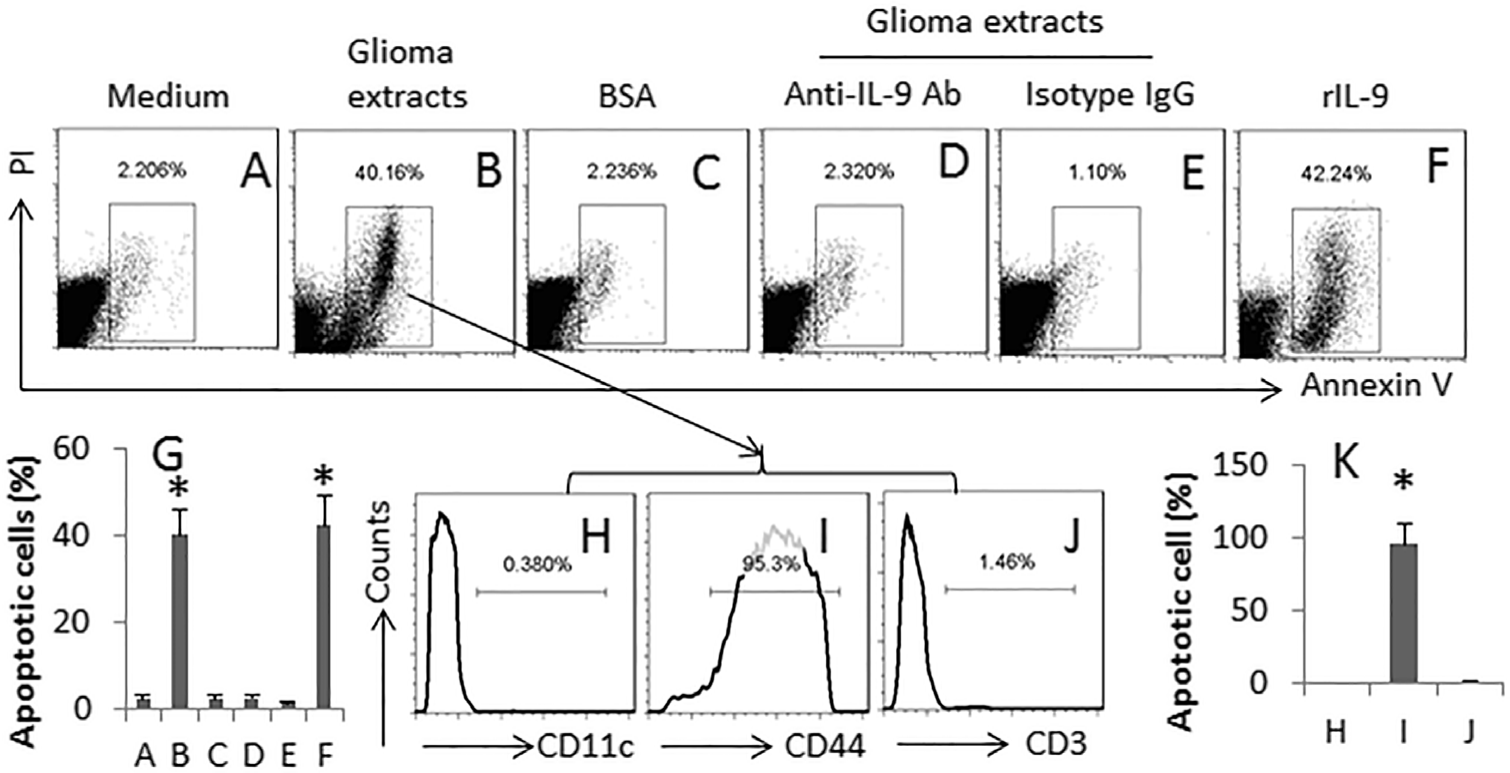
Glioma-specific Th9 cells induce glioma cell apoptosis Th9 cells were generated in the same procedures of Figure 4D. The cells (contain both Th9 cells and DCs; or #, naïve CD4^+^ T cells) were cultured with GL261 cells (the glioma cell line) at a ratio of 1:1 for 24 h. Anti-IL-9 Ab (500 ng/ml). The cells were analyzed by flow cytometry. (**A**–**F**) the gated dot plots indicate the apoptotic cells. (**G**) the bars indicate the summarized data of A-F. (**H**–**J**) the histograms indicate the phenotypes of the apoptotic cells in the gated cells of panel B. CD44 is a marker of glioma cells. (**K**) the bars indicate the summarized data of H-J. rIL-9: 100 ng/ml. The data of bars are presented as mean ± SD. *p < 0.01, compared with group A (panel G), or group G (panel K). The data are representatives of 3 independent experiments.

Th9 cells are capable of killing cancer cells [15]. We wondered if the generated specific Th9 cells were able to kill glioma cells. Thus, we cultured the glioma-specific Th9 cells (or naïve CD4^+^ T cells) and GL261 cells together in the presence of DCs. As shown by flow cytometry assay, the glioma-specific Th9 cells, but not the naïve CD4^+^ T cells, markedly induced GL261 cell apoptosis (Figure 6A–6C); the apoptosis was abolished by the presence of anti-IL-9 antibody (Figure 6D–6E). The presence of rIL-9 in the culture also induced the apoptosis of GL261 cells (Figure 6F). The apoptotic cells expressed the glioma marker CD44 (Figure 6H–6K), indicating the cells are glioma cells.

### SEB enhances the effects of immunotherapy on glioma growth

**Figure 7:**
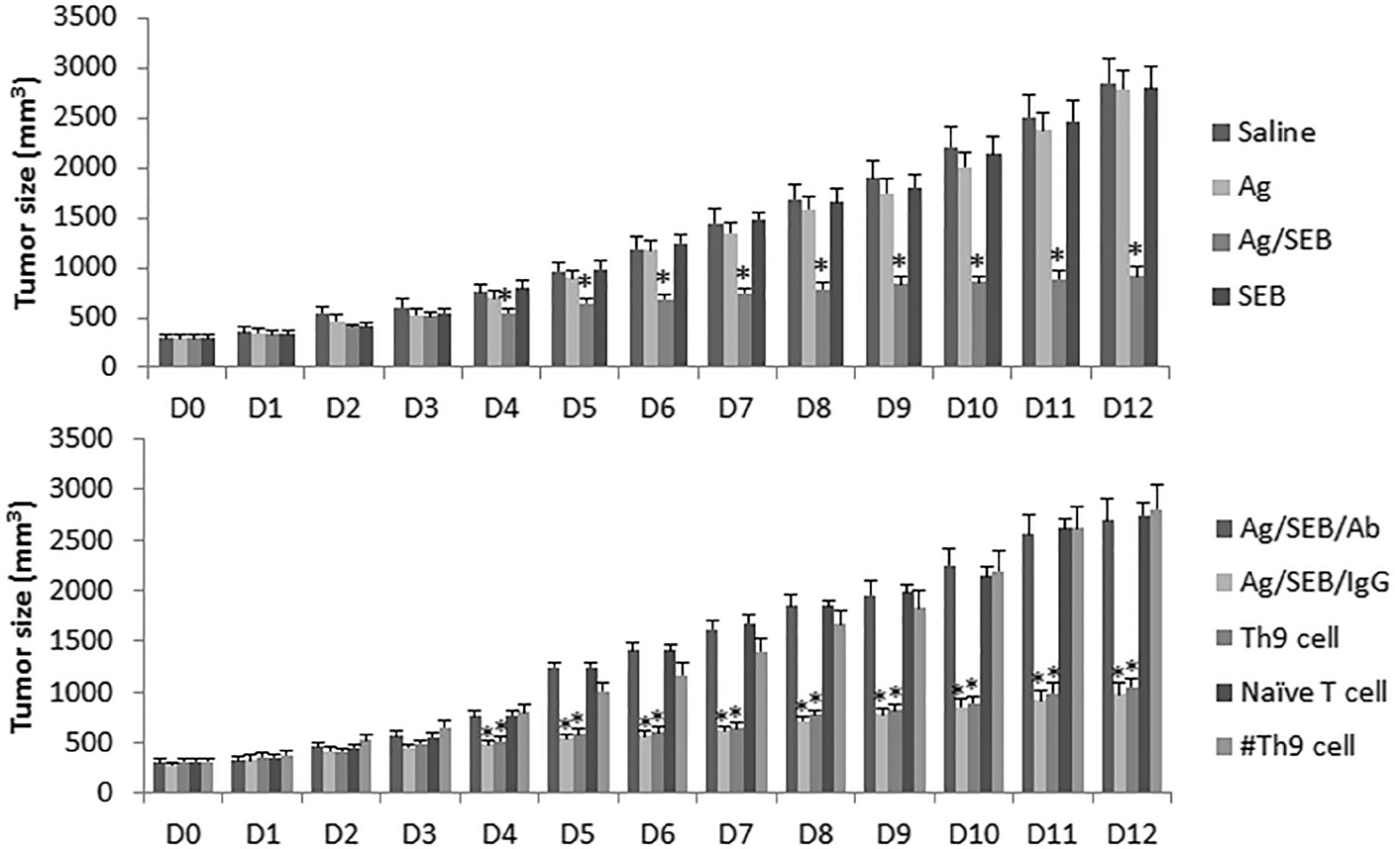
Administration with SEB enforces the effect of immunotherapy of Ag on experimental glioma The bars indicate the tumor size (mean ± SD) recorded from glioma-bearing mice. The treatment is denoted on the right side of the figure. Ag: GL261 cell extracts (100 μg/mouse; ip, daily). SEB (2 μg/mouse; ip; daily). Ab: Anti-IL-9 neutralizing Ab (200 μg/mouse; ip, day 3, 6 and 9). IgG: Isotype IgG (200 μg/mouse; ip, day 3, 6 and 9). Th9 cell (or naïve T cell, or #Th9 cell): Mice were adoptively transferred with glioma-specific Th9 cells (or naïve CD4^+^ T cells, or BSA-specific Th9 cells) at 10^6^ cell/mouse on day 3 and day 7 respectively. The bars indicate the tumor size. *p < 0.01, compared with the saline group. Each group consists of 9 mice.

Based on the fact that immunotherapy has been employed in the treatment of glioma [16], SEB can regulate skewed immune responses [17] and increases the development of Th9 cells as shown by the present data, we inferred that SEB might be able to enhance the immunotherapeutic effects on glioma. To test the inference, we developed a glioma-carrying mouse model. The glioma mice were treated with Ag (glioma cell extracts; using as a glioma specific Ag) or/and SEB. The results showed that the tumor growth was significantly inhibited in the mice treated with both Ag and SEB, but no measurable inhibitory effects of the tumor growth in mice treated with either Ag alone or SEB alone (Figure 7).

The present data show that SEB can facilitate the development of Th9 cells. Previous reports indicated that Th9 cells could suppress tumor growth [18]. We inferred that Th9 cells might play an important role in the glioma inhibition in the present study. To test this, we treated the glioma-bearing mice with anti-IL-9 Ab together with Ag/SEB. Indeed, the antitumor effects of Ag/SEB were abolished. To corroborate the results, we adoptively transferred the generated glioma-specific Th9 cells to the glioma-bearing mice; the glioma growth was significantly inhibited, whereas adoptively transferred with naïve CD4^+^ T cells did not show such an effect (Figure 7). In addition, no appreciable side effects were induced by the therapy of Ag/SEB in the glioma-bearing mice.

## DISCUSSION

Glioma is a common malignant tumor in the brain. The therapeutic effects on glioma are still poor currently. Thus, the invention of novel and effective therapeutic remedies for the treatment of glioma is of significance. The present data showed that administration with both SEB and glioma Ags had inhibitory effects on glioma growth in mice. Exposure of naïve CD4^+^ T cells to SEB triggered IL-9 gene transcription and IL-9 protein expression in the T cells.

One of the anatomical features of glioma is its diffuse distribution in the brain, which makes glioma refractory to be radically removed. Thus, additional therapies are usually required before or after surgery [19]. Immunotherapy is one of the options [3]. Bonomi et al. reported that human CD14^+^ cell-derived DCs primed by Paclitaxel strongly inhibited proliferation of U87 MG cells [20]. Several Toll like receptor agonists have been used in glioma clinic and achieved survival benefit in patients with glioma [21]. Our results provide a possible novel remedy to be used in the inhibition of glioma growth. By administration with SEB and glioma extracts, the glioma-specific Th9 cells were induced, which have a strong inhibitory effect on glioma growth. The data show that glioma specific CD4^+^ IL-9^+^ T cells were induced in glioma-bearing mice after treating with SEB and glioma extracts, but not in those treated with either SEB alone or glioma extracts alone. This phenomenon may be explained as that two signals are required to activate T cells. SEB can be one signal; the specific antigen, here is the glioma extracts, can be another signal. Thus, besides inducing IL-9 expression, SEB also functions as a T cell activator [22]. Together with glioma extracts (the specific Ag), SEB facilitates the generation of glioma specific CD4^+^ IL-9^+^ T cells. The detail mechanism needs to be further investigated.

It is suggested that Th9 cells are capable of inhibiting cancer growth [15]. Lu et al. indicate that Th9 cells elicit strong host antitumor CD8^+^ cytotoxic T cell responses by promoting Ccl20/Ccr6-dependent recruitment of DCs to the tumor tissues [18]. Th9 cells also have antitumor effect on promoting producing IL-3 to up regulate the expression of anti-apoptotic protein Bcl-XL, activate the p38, ERK and STAT5 signaling pathways [6], or induce IL-21 [15]. Our data emphasize the importance of Th9 cell's antitumor ability by showing that the administration of anti-IL-9 antibody abolished the glioma inhibitory ability of Th9 cells.

Multiple factors are involved in the induction of Th9 cells. Vegran et al. report that IL-1β induces phosphorylation of the transcription factor STAT1 and IRF1; the latter bind to the promoters of *Il9* and *Il21* to initiate the expression of IL-9 and IL-21 in Th9 cells [15]. STAT6, GATA3, and IRF4, are required for the differentiation of Th9 cells [23, 24]. It is suggested that PU.1 plays a critical role in generating Th9 cells [25]. Our data are in line with these previous studies by showing that exposing to SEB increases PU.1 activities that is associated with the expression of IL-9 in CD4^+^ T cells. SEB is a superantigen; it can activate T cells in collaborating with DCs. Previous reports indicate that SEB facilitates the initiation of Ag specific Th2 response [9]. IL-4 is a signature cytokine in the skewed Th2 polarization; it also plays a role in the generation of Th9 cells [10]. Jin et al. reports that administration with SEB induces higher expression of TGF-β in the nasal mucosa [26] while TGF-β is also involved in the induction of Th9 cells [27]. Although Th17 cells also produce IL-9 [28], our data show that the SEB/glioma extract-induced IL-9^+^ T cells are not Th17 cells, supporting the notion of the multiple sources of IL-9.

The results show that, after exposure to SEB, the phosphorylated p300 levels are up regulated in the CD4^+^ T cells. The pp300 acetylated the histone H3K9 at the IL-9 promoter locus. PU.1 is a transcription factor of IL-9. The results are in line with previous reports [29]; Busbee et al. indicate that the phosphorylated p300 binds PU.1 and initiates the IL-9 gene transcription. Ramming et al. also suggest that histone modifications in the PU.1 promoter regulate Th9-cell development [30].

The data show that, upon exposure to specific antigens, the glioma specific Th9 cells release IL-9 to induce glioma cell apoptosis. Similar findings were also reported previously. Fang et al. found that IL-9 induced melanoma cell apoptosis via increasing caspase-3 and pro-apoptotic molecule TRAIL [31]. Vegran et al. found that Th9 cells exerted potent anticancer functions in an IRF1- and IL-21 dependent manner [15]. It seems that IL-9 induces cancer cell apoptosis via various pathways; it may depend on cell types.

In summary, the present study shows that exposure to SEB and glioma protein extracts induces glioma specific Th9 cells. The Th9 cells produce IL-9 to induce glioma cell apoptosis and inhibit the tumor growth.

## MATERIALS AND METHODS

### Reagents

The ELISA kits of IL-4 (Cat#M4000B), IL-9 (DY409), IFN-γ (MIF00) and recombinant IL-9 were purchased from R&D Systems (Minneapolis, MN). SEB (s4881), garcinol (g5713), Annexin V kit, ChIP kit and protein G agarose beads were purchased from Sigma Aldrich (St. Louis., MO). The fluorochrome labeled antibodies of CD11c (561241), CD44 (563058), CD3 (561824), CD4 (563933), IL-9 (D9302C12), IL-4 (553047), IL-17 (560489) and IFN-γ (557998) were purchased from BD Biosciences (Franklin Lakes, NJ). The reagents for RT-qPCR and Western blotting were purchased from Invitrogen (Carlsbad, CA). Antibodies of H3K4ac, IL-9 (7923), p300 (585), pp300 (130210) and PU.1 (390405) were purchased from Biomart (Shanghai, China). The immune cell isolation kits were purchased from Miltenyi Biotech (San Diego, CA). The APAAP reagent kit was purchased from the Gukang Biotech (Guangzhou, China). The shRNA kits of VDR and p300 were purchased from Santa Cruz Biotech (Santa Cruz, CA).

### Mice

C57BL/6 mice (male, 6-8 week old) were purchased from the Guangzhou Experimental Animal Center. Mice were kept at a constant temperature of 23°C, relative humidity of 55 ± 5% and under a regular cycle (light: dark = 12 h: 12 h). The mice were allowed to access water and food freely. Mice were acclimatized for 7 days before using in the experiments. The using animal in the present study was approved by the Animal Ethic Committee at Guilin Medical University. The procedures were performed in accordance with the guidelines.

### Glioma cell culture

GL261 cells (a mouse glioma cell line; purchased from the Beijing Cell Bank, Beijing, China) and NG108-15 cells (another mouse glioma cells line) were cultured The cell lines were grown in DMEM medium supplemented with 10% fetal bovine serum (FBS) and 2 mM L-glutamine, 100 U/ml penicillin, 0.1 mg/ml streptomycin. The cells were incubated in a humidified atmosphere with 5% CO_2_ at 37°C. Before using in further experiments, the viability of the cells was greater than 99% as checked by Trypan exclusion assay.

### An *in vivo* tumor model

C57BL/6 mice were used in the present study. Forty-five mice were randomly divided into 5 groups. GL261 cells (10^6^ cells/mouse) were subcutaneously injected into the groin of the mice. The tumor size was recorded daily with a slide caliper, and converted to mm^3^ by the formula of (π/6 × length × width × height).

### Immunotherapy of tumor-bearing mice

The tumor-bearing mice were treated with Ag (the GL261 cell extracts), or SEB, or Ag and SEB. To test the role of IL-9 in the therapy, a group of tumor-bearing mice was treated with Ag/SEB/anti-IL-9 antibody (or isotype IgG). Another strategy was to adoptively transfer with glioma-specific Th9 cells (or naïve CD4^+^ T cells, or BSA-pulsed Th9 cells). The detail procedures are presented in Table 1.

**Table 1:** Immunotherapy procedures used in tumor-bearing mice

Group	Therapy	Description
1	Saline	0.3 ml/mouse, i.p., daily
2	Ag (GL261 cell extracts)	100 μg/mouse, i.p., daily
3	Ag/SEB	Ag (100 μg)/SEB (2 μg)/mouse, i.p., daily
4	SEB	2 μg/mouse, i.p., daily
5	Ag/SEB/anti-IL-9 Ab	Ag (100 μg)/SEB (2 μg)/mouse, i.p., dailyAnti-IL-9 Ab 200 μg/mouse, i.p., d3, d6, d9
6	Ag/SEB/isotype IgG	Ag (100 μg)/SEB (2 μg)/mouse, i.p., dailyIsotype IgG 200 μg/mouse, i.p., d3, d6, d9
7	Glioma-specific Th9 cell	10^6^ cells/mouse, tail vein injection, d3, d9
8	Naïve CD4^+^ T cell	10^6^ cells/mouse, tail vein injection, d3, d9
9	BSA-pulsed Th9 cell	10^6^ cells/mouse, tail vein injection, d3, d9

Ag = Antigen (GL261 cell extracts). i.p. = intraperitoneal injection. d = day.

Each group consists of 9 mice.

### Immunohistochemistry (APAAP method)

Glioma tissue was processed for cryosections. The sections were fixed with cold acetone for 30 min, treated with 0.1% H_2_O_2_, 1 mM levamisole and 5 mM MgCl_2_ for 30 min to quench endogenous peroxidase and phosphatase. After blocking with 1% BSA for 30 min, the sections were incubated with anti-CD4 and anti-IL-9 antibodies overnight at 4°C. After washing with PBS for 3 times, the sections were incubated with peroxidase-labeled and ALP (alkaline phosphatase)-labeled secondary antibodies for 1 h at room temperature. The sections were washed with PBS for 3 times and developed with DAB (diaminobenzidine) and FR/AS-MX respectively. The CD4 molecules were stained in brown and the IL-9 molecules were stained in rose pink. The sections were observed with a light microscope. The CD4^+^ IL-9^+^ cells were counted in randomly selected 20 fields per sample. All slides were coded. The observers were not aware of the code to avoid the observer bias.

### Protein extraction

Total proteins were extracted from the GL261 cells. The protein contents were determined by the Bio-Rad protein assay. The protein extracts were used as a specific glioma Ag (Ag) in further experiments.

### Immune cell isolation

The mice were sacrificed on day 12. The spleen and the GL261 cell tumor mass were removed; single cells were prepared following the published procedures [33]. Briefly, the tumor tissue was cut into small pieces and incubated with collagenase IV (0.5 mg/ml) at 37°C for 2 h with mild stirring. The cells were passed through a cell strainer (70 μm) and collected by centrifugation. The cells were then incubated with magnetic beads conjugated with antibodies of CD3 and CD4 by the magnetic cell sorting (MACS). The T cells were used in the flow cytometry assay. On the other hand, the CD25^+^ cells were selected out from the CD4^+^ T cells. The remained CD4^+^ CD25^−^ T cells were used in further experiments.

### Enzyme-linked immunosorbent assay (ELISA)

Cytokine levels were determined by ELISA with commercial reagent kits following the manufacturer's instructions.

### Flow cytometry

Cells were fixed with 2% paraformaldehyde for 2 h at room temperature. In case of intracellular staining, the fixatives contained 0.1% Triton X-100. The cells were blocked with 1% bovine serum albumin (BSA) for 30 min, and incubated with fluorochrome-labeled antibodies, or isotype IgG. After washing with phosphate buffered saline (PBS), the cells were analyzed with a flow cytometer (FACSCanto II, BD Bioscience, Shanghai, China). The data were analyzed with Flowjo. The data from isotype IgG stained cells were used as a gating reference.

### T cell proliferation assay

CD4^+^ CD25^−^ T cells were isolated from the spleen and glioma tissue by MACS, stained with CFSE, and cultured with DCs (T cell:DC = 10^5^:10^4^) in the presence of glioma cell extracts (5 μg/ml) for 3 days. The cells were analyzed by flow cytometry.

### Real time quantitative RT-PCR (RT-qPCR)

The cells were treated with TRIzol reagent to extract the total RNA. The cDNA was synthesized with a reverse transcription reagent kit. The qPCR was performed on a Mini Opticon real time PCR system (Bio-Rad, Shanghai, China) with the SYBR Green Master Mix. The results were calculated with the 2^−ΔΔCt^ method and normalized to a percentage of the internal control gene β-actin. The primers include: IL-9, forward, cttgcctgttttccatcggg; reverse, tctgtcttcatggtcggctt. β-actin, forward, gtgggaatgggtcagaagga; reverse, tcatcttttcacggttggcc.

### Western blotting

The total proteins were extracted from the cells, fractioned by SDS-PAGE (sodium dodecyl sulfate polyacrylamide gel electrophoresis) and transferred onto a polyvinylidene difluoride (PVDF) membrane. After blocking with 5% skim milk for 1 h at room temperature, the membrane was incubated with the primary antibodies (0.1 μg/ml) overnight at 4°C, and followed by incubation with the secondary antibodies (conjugated with horseradish peroxidase) for 1 h at room temperature. Washing with Tris-buffered saline-Tween 20 (TBST) was performed after each time of incubation. The immune blots on the membrane were developed with the enhanced chemiluminescence (ECL). The results were photographed with a KODAK Image Station 4000Pro (KODAK, Shanghai, China). The integrated density of the Western blots was determined by the software ImageJ (NIH, USA) and normalized to a percentage of the internal control protein of β-actin.

### Immunoprecipitation

The cell extracts were pre-cleared by incubating with protein G agarose beads for 1 h at 4°C. The supernatant was collected by centrifugation and incubated with antibodies of interest overnight at 4°C. The immune complexes were precipitated by incubating with protein G agarose beads for 2 h at 4°C. The beads were collected by centrifugation. The proteins on the beads were eluted with eluting buffer and analyzed by Western blotting.

### Chromatin immunoprecipitation (ChIP)

A commercial kit was used for the ChIP following the manufacturer's instruction. Briefly, the CD4^+^ T cells were fixed with 1% formaldehyde for 15 min and followed by sonication. The samples were pre-cleared by incubation with protein G beads for 1 h. After centrifugation, the antibody of PU.1 or pp300, or isotype IgG (control antibodies) and protein G was added to the supernatant for 16 h at 4°C. The beads were collected by centrifugation and washed with PBS for 3 times. The immune complexes on the beads were eluted by SDS buffer. The protein-DNA complexes were reversely separated. The DNA was extracted and processed by qPCR with a pair of IL-9 promoter primers of actgagttccagactcccgt and gcccagcacagaactgaaga (−435 to −56). The results were presented as relevant changes against the DNA input.

### Assessment of apoptotic cells

Cells were stained with propidium iodide (PI; 5 μg/ml, 5 min) and Annexin V reagent following the manufacturer's instructions. The cells were analyzed by flow cytometry. The Annexin V^+^ and PI^+^ Annexin V^+^ cells are regarded as apoptotic cells.

### Generation of Th9 cells

CD4^+^ CD25^−^ T cells were isolated from the mouse spleen by MACS with commercial reagent kits following the manufacturer's instructions. CD4^+^ CD25^−^ T cells and DCs (T cell:DC = 5:1) were cultured in the presence of SEB (100 ng/ml) and IL-2 (10 ng/ml) for 6 days with or without the presence of glioma cell extracts (5 μg/ml; or used BSA as a control Ag). The medium and related agents were changed in every 3-day. The cells were collected at the end of the culture and analyzed by flow cytometry.

### Adoptive transfer of Th9 cells

Th9 cells were injected to mice via the tail vein at 10^6^ cells/mouse in 0.1 ml saline. Control mice were injected with naïve CD4^+^ T cells.

### Statistics

Data are presented as mean ± SD. The difference between groups was determined by Student *t* test, or by ANOVA if more than two groups. A *p* < 0.05 was set as a significant criterion.

## SUPPLEMENTARY MATERIALS FIGURES


