# Supplementary material for: Induction of specific T helper-9 cells to inhibit glioma cell growth
Source: Oncotarget. 2016 Dec 16;8(3):4864–74. doi: 10.18632/oncotarget.13981 (PMC5354876; doi:10.18632/oncotarget.13981)
Supplement: Supplementary file 1 [file oncotarget-08-4864-s001.pdf]

Induction of specific T helper-9 cells to inhibit glioma cell growth

Supplementary Materials

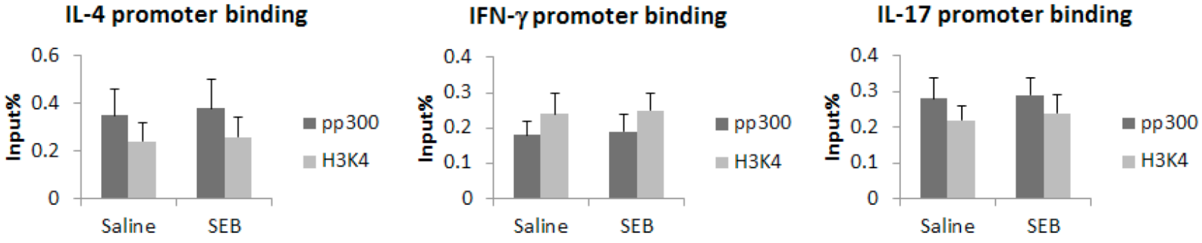

Supplementary Figure S1: Binding rate at promoters of IL-4, IFN-γ and IL-17 after exposure to SEB. $CD4^+$ $CD25^-$ T cells were isolated from the mouse spleen and cultured in the presence of saline or SEB (100 ng/ml) for 6 days. The cells were collected and analyzed by ChIP assay. The bars indicate the binding rate at the promoter loci of IL-4, IFN-γ and IL-17 by pp300 or H3K4. The data are summarized from data of 3 independent experiments. The primer sequences used in the experiments are listed below: IL-4: aacgaggtcacaggagaagg and caggacagagaaagcatcgc. IFN-γ: acgctccttctgaaagctct and gatggtgtcgttgaaggagc. IL-17: actctccaccgcaatgaaga and ctctcaggctccctcttcagttc.

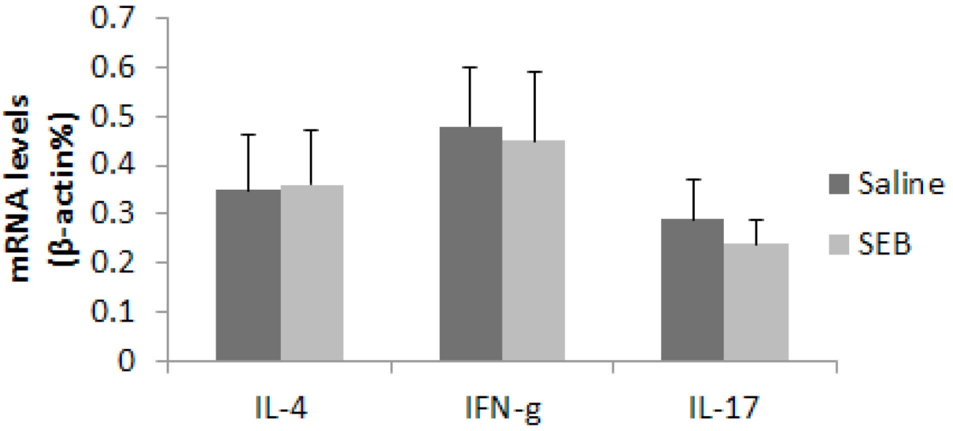

Supplementary Figure S2: mRNA levels of IL-4, IFN-γ and IL-17 after exposure to SEB. $CD4^+$ $CD25^-$ T cells were isolated from the mouse spleen and cultured in the presence of saline or SEB (100 ng/ml) for 6 days. The cells were collected and analyzed by RT-qPCR. The bars indicate the mRNA levels of IL-4, IFN-γ and IL-17 in the $CD4^+$ T cells. The primers used in the experiments include: IL-4: aacgaggtcacaggagaagg and tctgcagctccatgagaaca. IFN-γ: ttcttcagcaacagcaaggc and actccttttccgcttcctga. IL-17: actctccaccgcaatgaaga and ctctcaggctccctcttcag.
